# Rural-urban disparities in nutritional status among women in Ethiopia based on HIV serostatus: a cross-sectional study using demographic and health survey data

**DOI:** 10.1186/s12879-023-08490-8

**Published:** 2023-08-21

**Authors:** Hirut Abebe, Anette Agardh, Malachi Ochieng Arunda

**Affiliations:** Division of Social Medicine and Global Health, Department of Clinical Sciences, Lund University, Malmö, Sweden

**Keywords:** HIV, Nutritional status, Reproductive age women, Ethiopia

## Abstract

**Background:**

Ethiopia is one of the sub-Saharan African countries most affected by the human immunodeficiency virus (HIV) epidemic and also by severe undernutrition, which is particularly prevalent among women. HIV infection, reproductive biology, and their role in society increase the vulnerability of women to malnutrition. Various factors including urbanization could cause differences in the nutritional status of rural and urban residents. In this study, we aimed to assess rural-urban disparities in nutritional status among women of reproductive age based on HIV serostatus in Ethiopia.

**Method:**

Data from the Ethiopian Demographic and Health Survey (EDHS) conducted in 2016 were used. Among 15,683 women included in the survey, 8822 non-pregnant women aged 15–49 years, including those who gave birth two months before the DHS survey were included in this study. Multinomial logistic regression was used to determine the relative risk ratios (RRR) for the associations between study variables.

**Results:**

Generally, the prevalence of underweight among women of reproductive age was higher in rural residents (28.9%) than in urban residents (12.3%) in Ethiopia. Being overweight was more prevalent among urban women than rural women, (35.1% vs. 4.8%). About 32% of HIV-positive women were underweight in both rural and urban areas. About 29% of HIV-positive urban women were overweight compared to 3.4% of HIV-positive rural women. Among urban residents, HIV-positive women were about 4 times more likely to be underweight than their HIV-negative counterparts, RRR 3.8 (95% CI: 1.58, 9.26). However, there was no significant difference in nutritional status between HIV-positive and HIV-negative women living in rural areas. Anemic women were more likely to be underweight while, wealthy women were less likely to be underweight in both rural and urban areas. Women aged 25–49 years were generally more likely to be overweight/obese and less likely to be underweight compared to younger women aged $\le 24$years.

**Conclusion:**

Malnutrition was more prevalent among HIV-positive women living in urban Ethiopia. Targeted nutritional interventions for HIV-positive women of reproductive age living in urban areas could be considered. Furthermore, efforts should be made to improve the nutritional status of women of reproductive age across the country.

## Background

Nutrition is a fundamental pillar of human life and an essential driver of sustainable development. However, malnutrition in all its forms is a major health challenge that contributes to morbidity and mortality worldwide [1, 2]. According to the 2020 global nutrition report, most countries are experiencing a double burden of malnutrition, with undernutrition coexisting with overweight or obesity. However, undernutrition is 10 times more prevalent in low-income countries compared to high-income countries [3]. One out of every nine persons is undernourished, and one out of every three is overweight or obese worldwide. Over 613 million women of reproductive age (15–49 years) are affected by anemia due to iron and folic acid deficiencies [3].

The HIV epidemic has had a catastrophic impact on health, nutrition, and general socioeconomic development among severely affected countries such as Ethiopia [4]. The interaction between HIV infection and nutrition is complex and intertwined; malnutrition accelerates the progression of HIV infection, while HIV infection reduces individual food and nutrient intake [5].According to the UNAIDS 2021 report, an estimated 38.4 million people were living with HIV and 1.5 million were newly infected globally [6]. In this report, 54% of all people living with HIV were women and girls. In sub-Saharan African countries, young women aged 15–24 years are twice as likely to be living with HIV than men [6]. Ethiopia is one of the sub-Saharan African countries highly affected by the HIV epidemic, with about 610,000 people estimated to be living with HIV in 2021 [6].

People living with HIV have a greater need for energy due to increased resting energy expenditures and nutritional needs caused by HIV-related illnesses. However, the symptoms of HIV infection, as well as the presence of opportunistic infections, can reduce nutrient intake and absorption and lead to malnutrition [7, 8]. This bidirectional relationship between HIV and malnutrition affects the immune system and increases the vulnerability of people living with HIV. Reproductive biology, HIV infection, and their role in society increase the vulnerability of women to malnutrition, thus indicating a high risk for malnutrition among women living with HIV [2, 7]. Because of the potential health risks, it is vital to monitor women’s nutritional status, particularly in low-and middle-income countries. Various factors including reproductive biology, poverty, lack of education, and climatic conditions that affect food production could contribute to malnutrition among reproductive-aged women [2, 9]. There is evidence that differences in nutritional status between rural and urban residents are caused by urbanization and related factors, which include changes in dietary patterns, nutrient intake, and adoption of modern lifestyle [10].

Although studies have been conducted on HIV and nutritional status among adult individuals living with HIV in Ethiopia [11–13], no population-based study to our knowledge has investigated the rural-urban disparities in the nutritional status of reproductive-age women based on their HIV serostatus in Ethiopia. Thus, the aim of this study was to examine rural-urban disparities in nutritional status among women of reproductive-age based on HIV serostatus in Ethiopia. Further, we aimed to investigate factors associated with malnutrition among women of reproductive age in both (rural and urban) settings in Ethiopia.

## Materials and methods

### Study setting

Ethiopia is Africa’s second most populous country, divided into nine geographical regions and two federal-level administrative cities. Like most developing countries, Ethiopia’s economy is based on agriculture [14]. The population of Ethiopia was estimated to be 102 million in 2016, with a fertility rate of 4.46 and a gross domestic product per capita of 717.1 US$ [15]. According to the 2016 Demographic Health Survey, 22% of reproductive-age women were underweight [16]. In Ethiopia, about 610,000 people were estimated to be living with HIV in 2021 [6]. The Ethiopian government introduced free Antiretroviral Therapy (ART) in 2005, and nutrition is an integral component of comprehensive HIV care and support [17].

### Study design and data source

We used cross-sectional data from the Ethiopian Demographic and Health Survey (EDHS) conducted in 2016. The data were downloaded from the DHS program database after obtaining permission. The survey was conducted through a two-staged stratified cluster sampling design across the entire country and is nationally representative. In this national survey, a total of 15,683 women were included. The biomarker survey (anemia testing and HIV testing) was conducted using standard procedures and protocols to ensure anonymity and compliance with international and national ethical standards for research involving human subjects. Non-pregnant women aged 15–49 years, including those who gave birth two months before the 2016 DHS survey, and who had conclusive HIV test results were included in the study. Hence, a total of 8822 women were selected from the national survey and included in the analysis. For this study, the socio-demographic information, anthropometric measurements, and HIV serostatus were used from the merged datasets (HIV and women’s individual datasets).

### Study variables

#### Outcome variable

The dependent variable in this study was women’s nutritional status measured by body mass index (BMI) and classified as “underweight” (BMI ≤ 18.49 kg/m^2^), “normal weight” (BMI 18.5-24.99 kg/m^2^), “overweight” (BMI 25-29.99 kg/m^2^) and “obesity” (BMI ≥ 30 kg/m^2^). However, due to the small numbers in the obesity stratum, the last two categories were merged as “overweight”.

#### Independent variables

Participants’ HIV serostatus was the main predictor variable in this study and was dichotomized as HIV-positive and HIV-negative. Other independent variables included in the model were anemia and sociodemographic variables. Based on previous research on HIV and nutrition in sub-Saharan African countries [18–20], participants’ anemia status, marital status, age, sex of household head, wealth index, parity, and educational level variables were included in the model as explanatory variables or potential confounders to the association between HIV serostatus and nutritional status. Regarding anemia, hemoglobin concentration below 120 g/L was considered anemia. Information on anemia was obtained from collected blood samples and dichotomized as “not anemic” and “anemic” [21]. Marital status referred to both single and married women; single women were those who had never been married, widowed, divorced, or separated, while married women were those who were married or living together with a partner at the time of the survey. The women’s educational levels were categorized as no education, primary, secondary, and high education. The age of the women was categorized as younger (≤ 24 years old), 25–34 years old, and ≥ 35 years old. The household head was dichotomized as male and female-headed households. The wealth index (determined based on the number and kinds of consumer goods they own) of the women was categorized as poor, middle, and rich [16]. Maternal parity (total number of childbirth) was categorized as ≤ 3 and ≥ 4. Urban areas were defined as localities with 2000 or more residents and primarily engaged in non-agricultural activities, while rural areas comprise all areas not categorized as urban [16].

### Data analysis

Statistical analysis was performed using IBM SPSS Statistics, Version 27.0. Descriptive statistics, frequencies, and proportions were used to describe the general characteristics of the participants and the distribution of participants across the study variables by nutritional status and place of residence. Multinomial logistic regression analysis was conducted to examine the associations between HIV status, sociodemographic variables, anemia, and nutritional status (BMI categories) of women, stratified by place of residence. Independent variables were mutually adjusted for each other. Normal BMI was used as a base category and the relative risk ratios (RRR) were determined with a 95% confidence interval (CI).

### Ethical consideration

Permission was obtained from the DHS program to access and use the data for this study. The data used in this study were aggregated secondary data with no personal identifying information. The DHS data collection procedure adhered to national and international ethical principles for medical research involving human subjects.

## Result

**Fig. 1:**
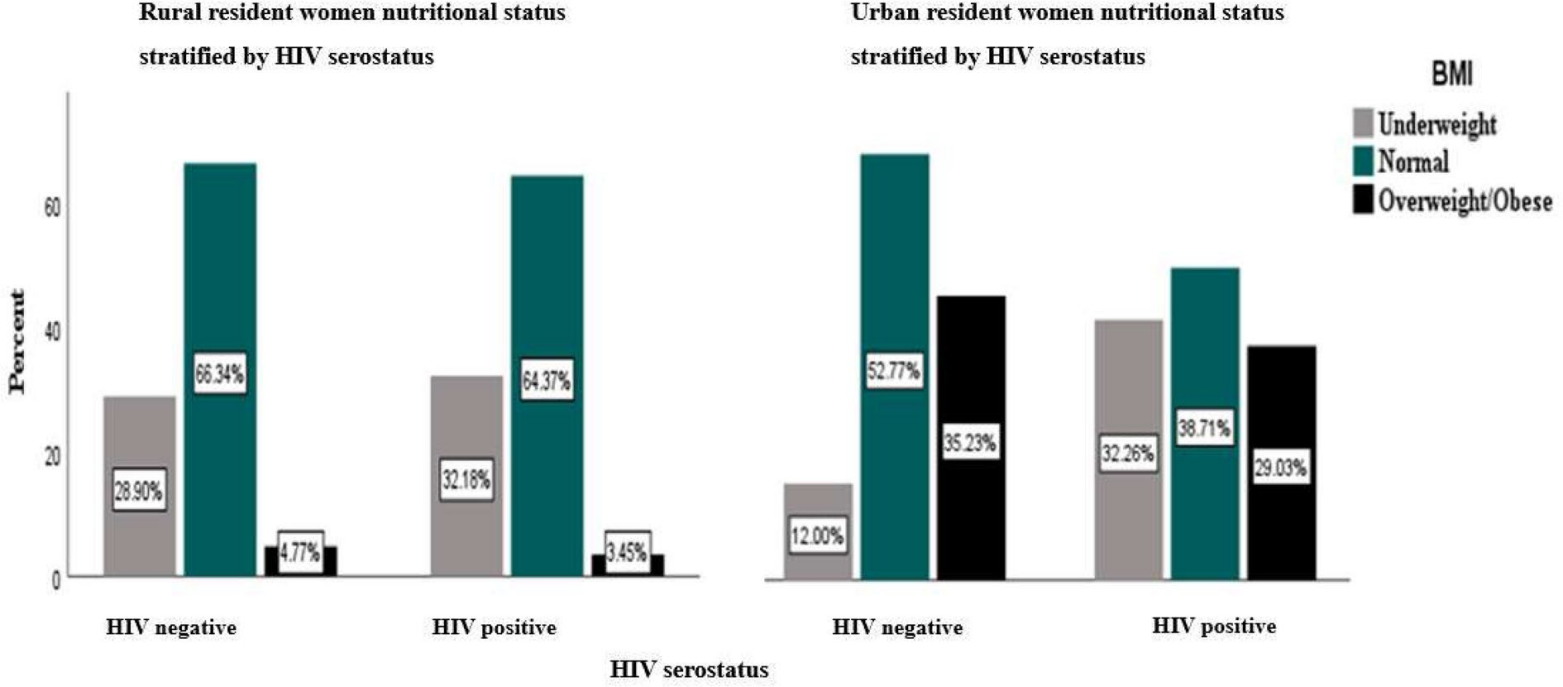
Distribution of BMI by HIV serostatus, stratified by place of residence among women of reproductive age in Ethiopia, using the 2016 Ethiopian Demographic and Health Survey data

Table 1; Fig. 1 show the distribution of HIV serostatus and sociodemographic variables by BMI. A total of 8822 participants were included in this study; the mean age of the study participants was 32.6 years (± 8.1SD). The prevalence of HIV among reproductive age women in rural and urban Ethiopia was 1.5% and 1.4%, respectively. Generally, the prevalence of underweight among reproductive aged women was higher among rural residents (28.9%) than among urban residents (12.3%). Being overweight was more prevalent among urban women in Ethiopia than among rural women (35.1% vs. 4.8%). Only 38.7% of HIV-positive women living in urban areas had normal body weight, compared to 64.4% of HIV-positive women living in rural areas. About 29.0% of HIV-positive women who were overweight lived in urban areas, while 3.4% lived in rural areas.

**Table 1:** Distribution of HIV serostatus, anemia status and sociodemographic factors by BMI, stratified by place of residence among women of reproductive age in Ethiopia, using the 2016 Ethiopian Demographic and Health Survey data, (N = 8822)

Variables		Rural			Urban		
		Underweight n(%)	Normal n(%)	Overweight n(%)	Underweight n(%)	Normal n(%)	Overweight n(%)
HIV Serostatus	HIV -ve	1745 (28.9)	4006 (66.3)	288 (4.8)	277 (12.0)	1218 (52.8)	813 (35.2)
	HIV + ve	28 (32.2)	56 (64.4)	3 (3.4)	10 (32.3)	12 (38.7)	9 (29.0)
	Total	1773 (28.9)	4062 (66.3)	291 (4.8)	287 (12.3)	1230 (52.6)	822 (35.1)
Parity	≥ 4	605 (29.0)	1386 (66.5)	93 (4.5)	94 (13.1)	395 (54.9)	230 (32.0)
	≤ 3	1168 (28.9)	2676 (66.2)	198 (4,9)	193 (11.9)	835 (51.5)	592 (36.5)
Anemia status	Anemic	623 (32.9)	1183(62.5)	86 (4.5)	86 (18.1)	259 (54.4)	131 (27.5)
	Not Anemic	1122 (27)	2824 (68.1)	202 (4.9)	194 (11.0)	931 (52.8)	638 (36.2)
Age (years)	≤ 24	384 (34.3)	708 (63.2)	29 (2.6)	80 (25.2)	197 (62.1)	40 (12.6)
	25–34	633 (26.0)	1676 (68.9)	122 (5.0)	118 (11.5)	584 (56.8)	327 (31.8)
	≥ 35	756 (29.4)	1678 (65.2)	140 (5.4)	89 (9.0)	449 (45.2)	455 (45.8)
Marital status	Single	230 (31.1)	468 (63.2)	42 (5.7)	79 (13.4)	316 (53.5)	196 (33.2)
	Married	1543 (28.6)	3594 (66.7)	249 (4.6)	208 (11.9)	914 (52.3)	626 (35.8)
Household head	Male	1317 (28.1)	3159 (67.5)	204 (4.4)	152 (11.4)	707 (52.9)	478 (35.8)
	Female	456 (31.5)	903 (62.4)	87 (6.0)	135 (13.5)	523 (52.2)	344 (34.3)
Wealth Index	Poor	1186 (33.7)	2202 (62.6)	132 (3.8)	28 (27.2)	66 (64.1)	9 (8.7)
	Middle	284 (24.6)	830 (71.9)	40 (3.5)	9 (25.7)	24 (68.6)	2 (5.7)
	Rich	303 (20.9)	1030 (70.9)	119 (8.2)	250 (11.4)	1140 (51.8)	811 (36.8)
Educational level	No education	1306 (29.2)	2960 (66.3)	199 (4.5)	84 (13.7)	365 (59.6)	163 (26.6)
	Primary	375 (26.5)	977 (69.1)	61 (4.3)	109 (12.7)	462 (53.8)	288 (33.5)
	Secondary	78 (38.2)	105 (51.5)	21 (10.3)	57 (11.0)	248 (48.1)	211 (40.9)
	Higher	14 (31.8)	20 (45.5)	10 (22.7)	37 (10.5)	155 (44.0)	160 (45.5)

**Fig. 2:**
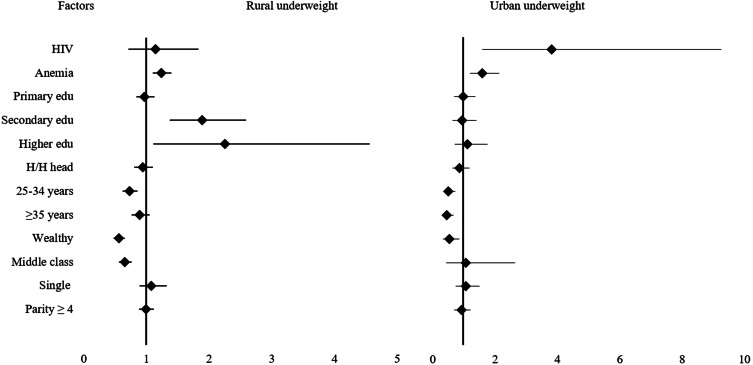
The forest plot presentation showing the Relative Risk Ratios (RRR) for the associations between predictors and underweight (BMI < 18.4) among women of reproductive age in Ethiopia using the 2016 Ethiopian Demographic and Health Survey data

**Fig. 3:**
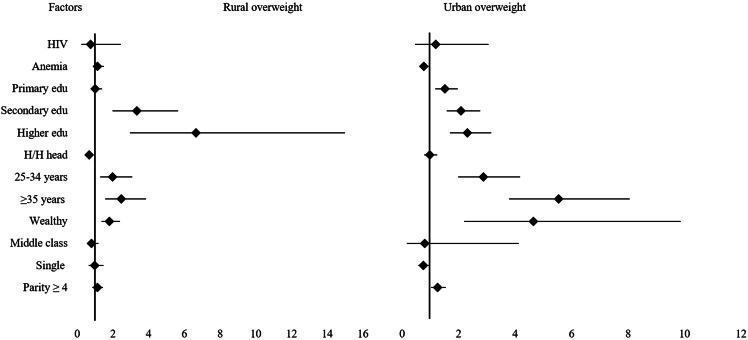
The forest plot presentation showing the Relative Risk Ratios (RRR) for the associations between predictors and overweight (BMI > 25) among women of reproductive age in Ethiopia using the 2016 Ethiopian Demographic and Health Survey data

Table 2 shows the relative risk ratios for the associations between HIV serostatus, anemia status, sociodemographic factors, and nutritional status. Urban resident HIV-positive women were almost 4 times more likely to be underweight compared to their HIV-negative urban counterparts, RRR 3.8 (95% CI: 1.58, 9.26). However, no significant difference was observed between HIV-positive and HIV-negative rural women. Among urban resident women, being anemic was associated with 1.6 times higher likelihood of being underweight and 23% less likelihood of being overweight, RRR 1.6 (95% CI 1.19, 2.13) and RRR 0.77 (95% CI 0.60, 0.98), respectively. Women aged 25–34 years and women aged ≥ 35 years living in urban areas were about 3–5 times more likely to be overweight. Wealthy urban women were about 50% less likely to be underweight and almost 5 times more likely to be overweight, RRR 4.65 (95% CI 2.19, 9.85). Women having primary, secondary, or high education were about 2 times more likely to be overweight, RRR ranged from 1.52 to 2.3 (95% CI 1.18 to 3.15). Single (never/formerly married) women were about 75% less likely to be overweight. The comparison between rural and urban women is summarized in Figs. 2 and 3.

**Table 2:** Multinomial logistic regression showing the Relative Risk Ratios for the associations between HIV serostatus, anemia status, sociodemographic factors and nutritional status (based on BMI) among women of reproductive age in Ethiopia using the 2016 Ethiopian Demographic and Health Survey data (N = 8822)

		Rural		Urban	
Variables		Underweight RRR (95% CI)	Overweight RRR (95% CI)	Underweight RRR (95% CI)	Overweight RRR (95% CI)
HIV Serostatus	HIV-positive	1.14 (0.71, 1.83)	0.75 (0.23, 2.44)	3.82 (1.58, 9.26) ^*^	1.18 (0.46, 3.06)
	HIV negative	1.00	1.00	1.00	1.00
Parity	≥ 4	0.99 (0.88, 1.12)	1.12 (0.86, 1.45)	0.92(0.69, 1.21)	1.26 (1.03, 1.55) ^*^
	≤ 3	1.00	1.00	1.00	1.00
Anemia status	Anemic	1.24 (1.10, 1.40) ^*^	1.14 (0.87, 1.49)	1.59 (1.19, 2.13) ^*^	0.77 (0.60, 0.97) ^*^
	Not Anemic	1.00	1.00	1.00	1.00
Educational Level	Primary	0.97 (0.84, 1.13)	1.01 (0.74, 1.38)	0.97 (0.69, 1.37)	1.52 (1.18, 1.97) ^*^
	Secondary	1.89 (1.37, 2.59) ^*^	3.34 (1.97, 5.66) ^*^	0.94 (0.63, 1.40)	2.08,(1.58, 2.76) ^*^
	Higher	2.25 (1,11, 4.56) ^*^	6.65 (2.95, 14.99) ^*^	1.11(0.70, 1.75)	2.31 (1.69, 3.15) ^*^
	No education	1.00	1.00	1.00	1.00
Household head	Male	0.94 (0.81, 1.10)	0.68 (0.50, 0.93) ^*^	0.86 (0.63, 1.17)	0.98 (0.78, 1.24)
	Female	1.00	1.00	1.00	1.00
Age (years)	25–34	0.73 (0.62, 0.86) ^*^	1.99 (1.29, 3.08) ^*^	0.50 (0.36, 0.71) ^*^	2.87 (1.98, 4.17) ^*^
	≥ 35	0.89 (0.76, 1.05)	2.46 (1.58, 3.85) ^*^	0.45 (0.31, 0.66) ^*^	5.53 (3.79, 8.05) ^*^
	≤ 24	1.00	1.00	1.00	1.00
Wealth index	Rich	0.56 (0.48, 0.65) ^*^	1.81 (1.37, 2.39) ^*^	0.53 (0.32, 0.85) ^*^	4.65 (2.19, 9.85) ^*^
	Middle	0.65 (0.56, 0.76) ^*^	0.81 (0.56, 1.18)	1.06 (0.42, 2.64)	0.80 (0.16, 4.13)
	Poor	1.00	1.00	1.00	1.00
Marital Status	Single	1.08 (0.89, 1.32)	0.97 (0.65, 1.46)	1.06 (0.74, 1.50)	0.75 (0.57, 0.97) ^*^
	Married	1.00	1.00	1.00	1.00

Normal weight: Reference for dependant variable. * P < 0.05

Among rural resident women, there was no significant difference in nutritional status between HIV-positive and HIV-negative women. Being anemic was significantly associated with being underweight; anemic women were 1.2 times more likely to be underweight, RRR 1.24 (95% CI 1.10, 1.40). Women aged 25–34 were about 25% less likely to be underweight and about 2 times more likely to be overweight, RRR 1.9 (95% CI 1.29, 3.08). Among rural women, middle-class and wealthy women were about 40% less likely to be underweight compared to poor women. Further, wealthy women in rural areas were about 2 times more likely to be overweight compared to poor women, RRR 1.8 (95% CI 1.37, 2.39). Having a secondary or high educational level was significantly associated with being overweight among rural resident women, RRR 3.34 (95% CI 1.97, 5.66) and RRR 6.65 (95% CI 2.95, 14.99), respectively. Furthermore, having a secondary or high education was about 2 times more likely to be associated with being underweight among rural resident women, RRR 1.89 (95% CI1.37, 2.59) and RRR 2.25 (95% CI 1,11, 4.56), respectively, compared to women having no education. In male-headed households, women were about 32% less likely to be overweight. The results of the multinomial regression analyses above are summarized in Table 2; Fig. 2, and Fig. 3.

## Discussion

To our knowledge, this is the first national study that examines rural-urban disparities in nutritional status among women of reproductive age in Ethiopia based on their HIV serostatus. In this study, we found that the prevalence of underweight among women was higher among rural residents than among urban residents. We also found that being overweight was more prevalent among urban women than among rural women. About 32% of HIV-positive women were underweight in both rural and urban areas. Urban HIV-positive women were almost 4 times more likely to be underweight than their HIV-negative urban counterparts. However, there was no significant difference in nutritional status between HIV-positive and HIV-negative rural women.

In this study, being underweight was found to be more prevalent among rural women than their urban counterparts. Similar results have been reported from different countries including Malawi, Nigeria, and South Africa indicating that being underweight was more prevalent among rural women [22, 23]. Previous studies have attributed this mainly to low socioeconomic status and unhealthy cultural practices, poor access to information regarding nutritional education, limited food security, lack of employment opportunities, and heavy household workload among other factors [3, 24]. According to the World Health Organization (WHO), the prevalence of underweight greater than 20% indicates the seriousness of the situation [25]. Our findings indicate that the prevalence of undernutrition among reproductive-age women in rural Ethiopia (28.9%) may thus represent a serious public health challenge.

The prevalence of undernutrition among rural and urban HIV-positive women was almost similar. Our findings showed that urban HIV-positive women had a higher prevalence of being overweight compared with their rural counterparts. This indicated the double burden of malnutrition, i.e., both underweight and overweight conditions found among HIV-positive women living in urban areas. In contrast to our findings, a meta-analysis conducted in 11 sub-Saharan African countries revealed that undernutrition was more prevalent among rural resident HIV-positive women than their urban HIV-positive counterparts [26]. In similarity to our findings, a study conducted by Takarinda et al. [27] in Zimbabwe revealed that people living with HIV faced the double burden of malnutrition. Currently, the double burden of malnutrition is an emerging public health concern, that might occur as the inevitable consequence of nutritional transition [28]. This has been observed in our findings, and it requires special nutritional evaluation and intervention.

In the present study, we found that urban resident HIV-positive women were about 4 times more likely to be underweight than their HIV-negative urban counterparts. A study conducted by Alebel et al. [29] indicated that in Ethiopia as in other sub-Saharan countries, HIV-positive individuals are more affected by undernutrition than the general population. Other studies conducted in Ethiopia also revealed that undernutrition is more prevalent among HIV-positive individuals [30, 31]. The above observed findings could be due to HIV-positive individuals having a higher need for energy and increased nutritional demands from HIV-related illnesses. The bidirectional relationship between HIV infection and nutrition reflects the fact that HIV infection reduces nutrient intake and absorption which leads to undernutrition, and conversely, that undernutrition exacerbates the progression of HIV infection [8]. As a result of this, the Ethiopian government acknowledged the problem of undernutrition and included nutritional intervention as one component of HIV care and support in the guidelines [17]. However, the higher likelihood of undernutrition among HIV-infected urban women highlighted by our findings indicates that efforts to prevent HIV infections among urban residents should be increased. Similarly, nutritional interventions for urban HIV-positive women should be further strengthened.

We found no significant difference in nutritional status between HIV-positive and HIV-negative rural women. This might be due to the high prevalence of undernutrition among the general population in rural Ethiopia [18]. Furthermore, the integration of nutritional intervention implemented in HIV care and support might contribute to the non-significant difference between HIV-positive and HIV-negative women in rural areas [17]. As stated above, the lack of significant difference in nutritional status between our study participants might not indicate the difference in micronutrient deficiency (hidden hunger), as micronutrient deficiency is more prevalent among HIV-positive individuals due to poor absorption [32].

It is reasonable to expect high malnutrition in rural areas due to lower socioeconomic status, lower nutritional awareness, lower availability and lower diversity of food, as well as inadequate health services. A study conducted in southern Ethiopia that aimed to assess the prevalence of malnutrition and its associated factors among adults people living with HIV/AIDS indicated that, compared with HIV-positive urban residents, rural resident HIV-positive individuals were 2 times more likely to be undernourished [33]. Furthermore, self-stigmatization and stigmatization by others may also increase the impact of HIV on health, making it more difficult to engage in economically productive activities, thus, limiting access to adequate and nutritious diets. However, the integration of nutritional intervention into HIV care and support might certainly contribute to reduced undernutrition among HIV-positive individuals [17].

We found that being anemic was significantly associated with the nutritional status of rural and urban resident women. Anemic women were more likely to be underweight and less likely to be overweight among rural and urban resident women. In line with our findings, a study conducted in Rwanda revealed that being underweight increased the odds of anemia, and being obese reduced the odds of being anemia among women of reproductive age [34]. Anemia is a complicated problem in which iron deficiency is the most prevalent proximate cause, which is more common among undernourished women [35].

Generally, women having formal education compared with those with no formal education were more likely to be overweight/obese among urban women. Similarly, rural resident women having secondary and high education were about 3–6 times more likely to be overweight when compared with women having no education. Previous studies conducted in low-income countries revealed that having an education was significantly associated with being overweight [19, 36–38]. This could be because women with higher education might have greater knowledge of how to overcome undernutrition. Also, due to their higher income and greater independence, they may live a life with less physical activity and higher access to energy-dense foods, which are considered to be causes of being overweight. On the other hand, women with lower education tend to work in labor-intensive jobs in low-income countries [39].

However, a noteworthy observation arises in rural areas: women having a secondary and high education tend to have a higher prevalence of underweight. It is crucial to acknowledge that educational accomplishments alone may not directly translate into improved nutritional status, especially in rural settings where there is a scarcity of job opportunities that require higher education. It is conceivable that women with advanced educational backgrounds in rural areas face specific barriers or challenges that contribute to their higher prevalence of underweight, factors that were not accounted for in our study. Further research is warranted to investigate the underlying factors that contribute to this observed association.

Overall, we also found that women aged 25 years or older were less likely to be underweight compared with those younger ($\le$24 years ) women among rural and urban resident women and more likely to be overweight compared with those younger women. In agreement with our findings, studies conducted in Ethiopia and India also indicated that BMI increased with age, while being underweight decreased as age increased [19, 40]. However, another study in Nepal indicated that there was no significant association between age and nutritional status among women of reproductive age [41]. This could be due to body composition changes throughout life and loss of muscle mass increase with age. Reduction of muscle mass (beginning at the age of 30) followed by an increase in adipose tissue could occur due to age-triggered changes in physical activity and hormonal changes, which favor overweight/obesity [42].

We found wealth status of the women was significantly associated with the nutritional status of rural and urban resident women. Among rural residents, middle-class, and wealthy women were less likely to be underweight compared with poor women and among urban residents, wealthy women were less likely to be underweight compared with poor women. On the other hand, urban and rural wealthy women were more likely to be overweight compared with poor women. In similarity to our findings, previous studies highlighted that a higher wealth status was significantly associated with being overweight among women of reproductive age [19, 43]. This could be related to many unhealthy cultural norms that favor overweight/obesity among women in low- income countries. Cultural practices related to food and physical activity might contribute to overweight/obesity. Furthermore, despite having better knowledge about healthy food and resources, wealthy women in low-income countries may encounter several hurdles to engage in physical activity and eating healthful foods [44].

Among rural residents, women living in a male-headed household were less likely to be overweight than women in a female-headed household. This could be because male-headed households may provide better opportunities to access better and healthy food consumption [45].

### Methodological considerations

A major strength of this study is that we used a large, nationally representative sample which increased the validity of the findings and generalizability to other similar settings. Further, our findings could be used as baseline information for further interventional studies and for program development in the prevention of malnutrition among women of reproductive age. However, the data used for this study were cross-sectional in nature, which limits the possibility to draw conclusions about the cause-and-effect relationship among study variables. Furthermore, the nutritional status assessment was solely based on BMI, and incorporating additional elements such as body fat, muscle mass, and other biochemical blood studies would have provided a more comprehensive assessment of participants’ nutritional status. We cannot determine whether any disparities in nutritional status found between HIV-positive and HIV-negative women are due to HIV since we do not know when women contracted HIV or became malnourished. The survey does not directly interview individuals about their HIV status and ART use, due to ethical reasons. Another limitation of the study was that the use of ART could lead to lipodystrophy and increases in BMI among ART users.

## Conclusion

Malnutrition was more prevalent among HIV-positive women living in urban Ethiopia. Targeted nutritional interventions for HIV-positive women of reproductive age living in urban areas could be considered. Furthermore, efforts should be made to improve the nutritional status of women of reproductive age across the country.

## Data Availability

The dataset used in this study is available in the demographic health and survey repository in http://dhsprogram.com/data/.
